# Observational study on the application of different esketamine gargle doses for the prevention of postoperative sore throat after laryngeal mask airway insertion

**DOI:** 10.3389/fphar.2026.1882856

**Published:** 2026-08-07

**Authors:** Le Bo, Yi Chen, Linsen Sun, Peng Zhai, Yan Jiang, Xiaoxiao Xu, Xiangsheng Xiong

**Affiliations:** 1 Department of Anesthesiology, Huai’an Hospital Affiliated to Yangzhou University (The Fifth People’s Hospital of Huai’an), Huaian, China; 2 Department of Anesthesiology and Perioperative Medicine, General Hospital of Ningxia Medical University, Yinchuan, China

**Keywords:** esketamine, gargle, laryngeal mask airway, observational study, postoperative sore throat

## Abstract

**Background:**

Ketamine gargle is considered a highly effective measure for preventing postoperative sore throat (POST). This randomized controlled study aimed to explore the efficacy, safety, and optimal dosage of esketamine gargle for preventing POST following laryngeal mask airway insertion.

**Methods:**

A total of 150 patients were randomly assigned to receive normal saline (NS), 25 mg, or 50 mg of esketamine gargle at a 1:1:1 ratio for 30 s prior to general anesthesia. The primary outcomes were the incidence of POST at 2, 6, and 24 h postoperatively. The secondary outcomes included the severity of POST at 2, 6, and 24 h postoperatively, along with the incidence and severity of dysphagia and hoarseness. Other outcomes included the incidence of the first intubation attempt, repositioning during surgery, convert to tracheal intubation, SpO_2_ < 90%, blood stain on the laryngeal mask airway, tachycardia and hypertension following gargling, intraoperative peak airway pressure, and end - tidal carbon dioxide partial pressure (PetCO_2_).

**Results:**

The incidence of POST at 2, 6, and 24 h postoperatively was 32%, 10%, and 12%; 22%, 8%, and 8%; and 12%, 6%, and 4%, respectively, with a statistically significant difference among groups at 2 h postoperatively (*P* = 0.007). In comparison with the NS group, the incidence of POST at 2 h postopertively in the 25 mg group was significantly lower (32% vs. 10%, *P* = 0.013). No statistically significant differences were observed in the remaining outcomes.

**Conclusion:**

Esketamine gargle is effective and safe for preventing POST following laryngeal mask airway insertion, and a dose of 25 mg is recommended.

**Clinical Trial Registration:**

ChiCTR2500099787.

## 1 Introduction

Postoperative sore throat (POST) is one of the most prevalent complications subsequent to general anesthesia with endotracheal intubation. Although it may exhibit a certain degree of self - limitation, the unpleasant experience can impact patients’ satisfaction and postoperative recovery (Mukesh et al., 2025; Chen et al., 2025). The laryngeal mask airway is currently utilized more extensively and has been proven to be safe in laparoscopic surgery. It presents advantages compared to endotracheal intubation by enhancing circulatory and respiratory stability, minimizing the influence on mucociliary clearance, and reducing the necessary dosage of anesthetic agents (Liu et al., 2021). In laparoscopic abdominal surgeries, including gynecological operations, the specific Trendelenburg position, which involves the patient being in a head - down position, facilitates surgical procedures. However, it increases the pressure on the throat and leads to intraoperative and postoperative edema, thereby contributing to the occurrence of POST (Siktas et al., 2024; Wang et al., 2025).

Currently, measures to address POST, including humidifying and warming the tubing, utilizing low-pressure large-volume cuffs, altering the tubing material, and employing drugs for prevention and treatment, are being continuously reported in an attempt to further reduce its incidence and severity (Senol Celik et al., 2024; Guan et al., 2025). However, preoperative ketamine gargle and other pharmaceutical preventive treatments are considered highly effective measures for preventing POST (Tadesse et al., 2024). Compared with ketamine, esketamine exhibits higher analgesic efficacy and greater circulatory stability, accompanied by fewer psychiatric adverse effects (Feeney and Papakostas, 2023). Nevertheless, at present, there is a paucity of evidence concerning the utilization of esketamine gargle for preventing POST. This study aims to explore the efficacy, safety, and optimal dosage of esketamine gargle for preventing POST in patients undergoing laparoscopic abdominal surgery with laryngeal mask airway insertion.

## 2 Materials and methods

### 2.1 Study design

This randomized controlled study was conducted in accordance with the CONSORT 2025 Statement at the Huai’an Hospital Affiliated to Yangzhou University (Huai’an Fifth People’s Hospital), Jiangsu Province, China, from May 2025 to April 2026. The trial was registered with the Chinese Clinical Trial Registry on 28 March 2025 (ChiCTR2500099787). No important amendments to the trial protocol, statistical analysis plan, primary/secondary outcomes, or analytic strategies were implemented after trial initiation.

### 2.2 Sample and setting

Eligibility for inclusion was limited to females 18–60 years of age with an ASA grade of I or II who were undergoing laparoscopic abdominal surgery with laryngeal mask airway insertion. Patients were excluded with an anticipated difficult airway or a scheduled surgery duration outside the 1-to-4-h window; a documented allergy or contraindication to esketamine; pre-existing respiratory diseases (e.g., bronchitis, COPD); and chronic throat pain or inflammation prior to surgery.

Patients were randomly assigned to the NS group, the 25 mg esketamine group, and the 50 mg esketamine group in a 1:1:1 ratio using the random number table method. Prior to the patients’ entry into the operating room, a researcher randomly assigned them to the three groups mentioned above, and the assignment results were placed in an opaque box.

### 2.3 Intervention

This researcher prepared the drugs, specifically 30 mL of a colorless liquid (esketamine was diluted using normal saline) in a 50 - mL transparent and unmarked small container, and then transferred it to the operating - room nurse. The patients gargle with the beforehand colorless liquid provided by the operating - room nurse for 30 s. Neither the operating - room nurse, the anesthesiologist, nor the patients were blinded of the drugs.

Patients were admitted to the operating room and received standard anesthesia and nursing care. In a relaxed state, the mean value of three consecutive measurements of vital signs, including heart rate (HR), non-invasive blood pressure (NBP) and transcutaneous oxygen saturation (SpO_2_), taken at 2 - minute intervals, was employed as the baseline value for all patients. Given the hemodynamic effects of esketamine, the patients’ NBP was maintained within 20% of the baseline value. All patients underwent spontaneous ventilation with oxygen for 3–5 min, followed by the administration of general anesthesia. The anesthesia induction consisted of midazolam 0.05–0.1 mg/kg, sufentanil 0.1–0.2 μg/kg, etomidate 0.1–0.2 mg/kg, and rocuronium 0.6–0.8 mg/kg. After 3–5 min of manual ventilation, a laryngeal mask airway was inserted, with the size being selected by the anesthesiologist in charge.

Following the successful placement of the laryngeal mask airway and the initiation of ventilation, the adequacy of ventilation was ascertained through auscultation of bilateral lung breath sounds and observation of the end - tidal carbon dioxide partial pressure (PetCO_2_) waveform. General anesthesia was maintained via continuous intravenous infusion of propofol at a rate of 4–6 mg/kg/h and remifentanil at a rate of 0.05–0.3 μg/kg/min. Rocuronium was administered intermittently to sustain muscle relaxation. In the event that severe laryngeal mask airway leakage, high airway pressure accompanied by the tidal volume failing to reach the set value occurred after general anesthesia, the laryngeal mask airway was reinserted. If the aforementioned situation recurred persistently, the observation was terminated, and tracheal intubation was performed. Upon completion of the operation, the patient was transferred to the PACU for recovery.

### 2.4 Data collection

The primary outcomes were the incidence of POST at 2, 6, and 24 h postoperatively. The secondary outcomes included the severity of POST at 2, 6, and 24 h postoperatively, along with the incidence and severity of dysphagia and hoarseness. Other outcomes included the incidence of the first intubation attempt, repositioning during surgery, convert to tracheal intubation, SpO_2_ < 90%, blood stain on the laryngeal mask airway, tachycardia and hypertension (defined as heart rate >100 beats per minute and systolic blood pressure >120% of the baseline value) following gargling, intraoperative peak airway pressure, and PetCO_2_ were measured at 3 min, 30 min, 60 min, 90 min, 120 min after intubation, and before extubation.

### 2.5 Data analysis

According to the literature reports, the incidence of POST associated with the laryngeal mask airway was approximately 28.8% (Mohan et al., 2024). Assuming that the incidences of POST after gargling with 25 mg and 50 mg of esketamine were 10% and 7% respectively, the total sample size was calculated to be 134 cases through a comparison of multiple groups of rates, with a type - I error of 5% and a type - II error of 20%. Taking into account the dropout factors, an additional approximately 10% of the sample size was added to each group, and 50 patients were required to be included in each group. The trial would only be terminated prematurely by the principal investigator and institutional review board in cases of severe unexpected serious adverse events leading to obvious safety risks, without any quantitative statistical stopping thresholds.

Statistical analysis was carried out using SPSS version 25.0 (IBM SPSS, Inc., Chicago, IL, USA). The Shapiro - Wilk test was employed to evaluate the normality of continuous variables. One - way analysis of variance (ANOVA) was employed for data that followed a normal distribution, with post - hoc Bonferroni correction implemented for multiple comparisons. The Kruskal - Wallis test was employed for data that did not follow a normal distribution, with post - hoc Dunn correction implemented for multiple comparisons. Chi - square tests were employed for categorical variables, and pairwise comparisons were implemented subsequent to the discovery of a significant overall difference. A *P* value of less than 0.05 was regarded as statistically significant.

### 2.6 Ethical considerations

This study was approved by the Ethics Committee of Huai’an Hospital Affiliated to Yangzhou University (Huai’an Fifth People’s Hospital) (Approval No. KY-P-2025-04). Written informed consent was obtained from all participants prior to their involvement.

## 3 Results

### 3.1 Study participants

**FIGURE 1:**
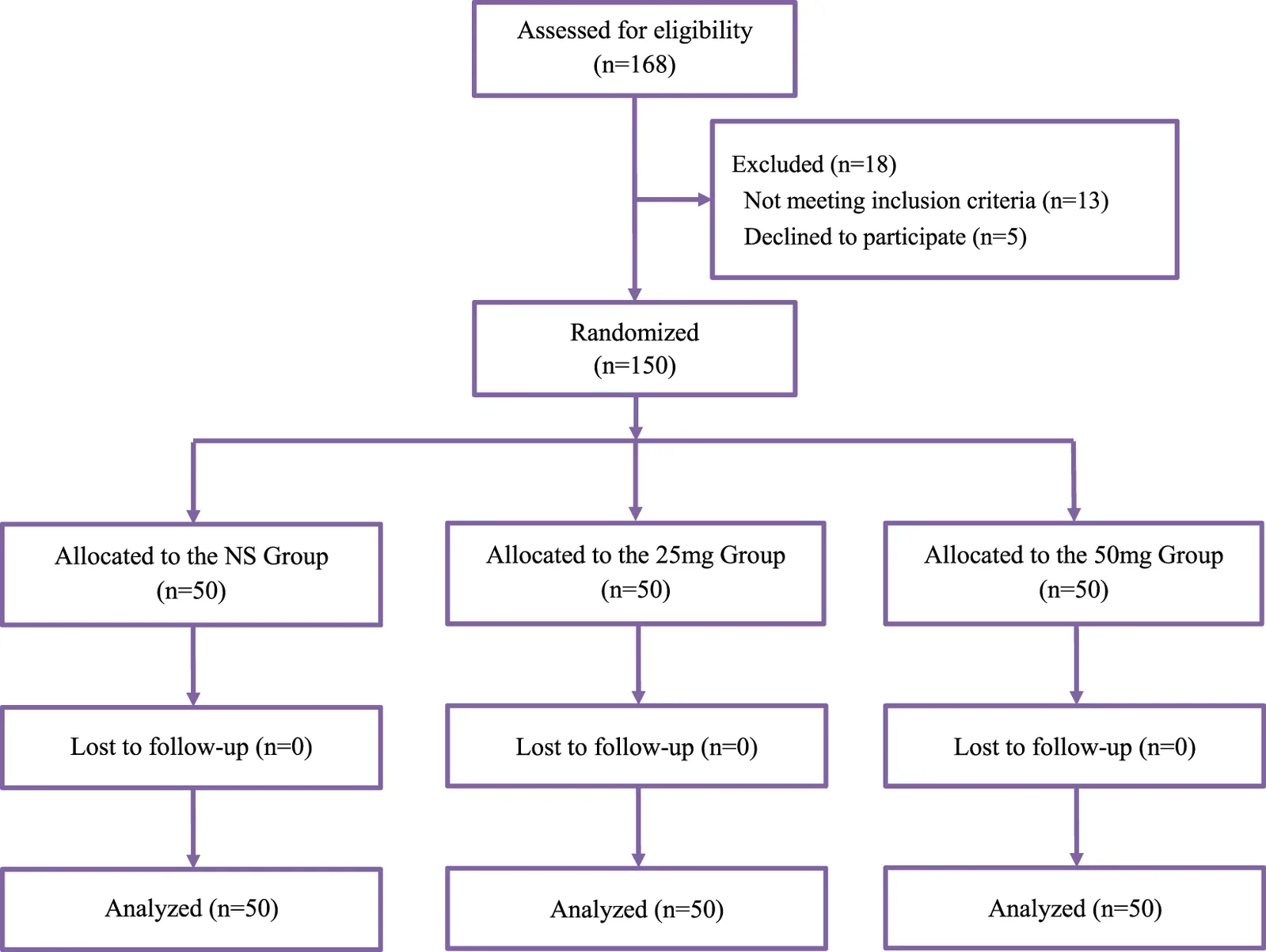
The flowchart.

A total of 150 patients were incorporated into the final analysis, with 50 cases allocated to each group. The flowchart depicting patient inclusion is presented in Figure 1. There were no statistically significant differences in the demographic and baseline characteristics of all patients, as shown in Table 1. No unintended events occurred in each group.

**TABLE 1:** Demographic and baseline characteristics.

Characteristics	NS group (n = 50)	25 mg group (n = 50)	50 mg group (n = 50)	*P* value
Age (years)	40.7 ± 11.5	43.5 ± 8.7	44.4 ± 10.1	0.167
Height (cm)	159.9 ± 5.2	159.4 ± 5.5	157.9 ± 4.8	0.131
Weight (kg)	61.3 ± 9.0	61.6 ± 7.4	61.6 ± 9.7	0.977
BMI (kg/m^2^)	23.9 ± 3.1	24.3 ± 2.8	24.7 ± 3.6	0.499
ASA grade	​	​	​	0.714
I, n (%)	5 (10%)	5 (10%)	3 (6%)	​
II, n (%)	45 (90%)	45 (90%)	47 (94%)	​
Mallampati grade, n (%)	​	​	​	0.878
I, n (%)	17 (34%)	18 (36%)	22 (44%)	​
II, n (%)	32 (64%)	31 (62%)	27 (54%)	​
III, n (%)	1 (2%)	1 (2%)	1 (2%)	​
Type of surgery	​	​	​	0.645
Uterine surgery, n (%)	36 (72%)	38 (76%)	40 (80%)	​
Ovarian surgery, n (%)	14 (28%)	12 (24%)	10 (20%)	​

Data are presented as mean ± SD, and number (%). One-way analysis of variance (ANOVA) and chi-square tests were employed for continuous variables with normal distribution and categorical variables, respectively.

### 3.2 Primary outcomes

The incidence and severity of POST at different time points following gargling with varying doses of esketamine are presented in Table 2. At 2 h postoperatively, gargling with 25 mg and 50 mg of esketamine effectively reduced the incidence of POST (*P* = 0.007). Among them, the incidence of POST in the 25 mg group was lower compared to that in the NS group (32% vs. 10%, *P* = 0.013). There were no statistically significant differences in the incidence and severity of POST among the three groups at other time points.

**TABLE 2:** Incidence and severity of postoperative sore throat.

Incidence and severity	NS group (n = 50)	25 mg group (n = 50)	50 mg group (n = 50)	P value
2 h postopertively				
POST, n (%)	16 (32%)^*^	5 (10%)	6 (12%)	0.007
Severity	​	​	​	0.865
Mild, n (%)	9 (56.2%)	2 (40%)	3 (50%)	​
Moderate, n (%)	3 (18.8%)	2 (40%)	1 (16.7%)	​
Severe, n (%)	4 (25%)	1 (20%)	2 (33.3%)	​
6 h postopertively				
POST, n (%)	11 (22%)	4 (8%)	4 (8%)	0.052
Severity	​	​	​	0.478
Mild, n (%)	9 (81.8%)	3 (75%)	2 (50%)	​
Moderate, n (%)	1 (9.1%)	1 (25%)	2 (50%)	​
Severe, n (%)	1 (9.1%)	0 (0.0%)	0 (0%)	​
24 h postopertively				
POST, n (%)	6 (12%)	3 (6%)	4 (8%)	0.555
Severity	​	​	​	0.296
Mild, n (%)	6 (100%)	3 (100%)	3 (75%)	​
Moderate, n (%)	0 (0%)	0 (0%)	1 (25%)	​
Severe, n (%)	0 (0%)	0 (0%)	0 (0%)	​

Data are presented as number (%). Chi-square tests were employed for categorical variables, and pairwise comparisons were implemented subsequent to the discovery of a significant overall difference.

* *P* = 0.013 vs. 25 mg group.

### 3.3 Secondary outcomes

As presented in Table 3, there were no statistically significant differences in the incidence and severity of dysphagia and hoarseness at different time points after surgery among the groups. Similarly, as shown in Table 4, there were no statistically significant differences in laryngeal mask airway - related outcomes, adverse events, intraoperative peak airway pressure, and PetCO_2_ among the groups. Importantly, there were no cases of re-insertion of the laryngeal mask airway or rescue intubation among the three groups.

**TABLE 3:** Incidence and intensity of dysphagia and hoarseness.

Incidence and severity	NS group (n = 50)	25 mg group (n = 50)	50 mg group (n = 50)	*P* value
2 h postopertively				
Dysphagia, n (%)	3 (6%)	3 (6%)	2 (4%)	0.876
Severity	​	​	​	0.386
Mild, n (%)	3 (100%)	2 (66.7%)	2 (100%)	​
Moderate, n (%)	0 (0%)	1 (33.3%)	0 (0%)	​
Hoarseness, n (%)	0 (0%)	1 (2%)	0 (0%)	0.365
Severity	​	​	​	1.000
Mild, n (%)	0 (0%)	1 (100%)	0 (0%)	​
Moderate, n (%)	0 (0%)	0 (0%)	0 (0%)	​
6 h postopertively				
Dysphagia, n (%)	2 (4%)	1 (2%)	2 (4%)	0.813
Severity	​	​	​	1.000
Mild, n (%)	2 (100%)	1 (100%)	2 (100%)	​
Moderate, n (%)	0 (0%)	0 (0%)	0 (0%)	​
Hoarseness, n (%)	0 (0%)	1 (2%)	0 (0%)	0.365
Severity	​	​	​	1.000
Mild, n (%)	0 (0%)	1 (100%)	0 (0%)	​
Moderate, n (%)	0 (0%)	0 (0%)	0 (0%)	​
24 h postopertively				
Dysphagia, n (%)	1 (2%)	0 (0%)	1 (2%)	0.602
Severity	​	​	​	1.000
Mild, n (%)	1 (100%)	0 (0%)	1 (100%)	​
Moderate, n (%)	0 (0%)	0 (0%)	0 (0%)	​
Hoarseness, n (%)	0 (0%)	1 (2%)	0 (0%)	0.365
Severity	​	​	​	1.000
Mild, n (%)	0 (0%)	1 (100%)	0 (0%)	​
Moderate, n (%)	0 (0%)	0 (0%)	0 (0%)	​

Data are presented as number (%). Chi-square tests were employed for categorical variables.

**TABLE 4:** Factors associated with laryngeal mask intubation and adverse effects.

Factors	NS group (n = 50)	25 mg group (n = 50)	50 mg group (n = 50)	*P* value
Duration of anesthesia (min)	150 [118, 188]	149 [130, 189]	150 [123, 180]	0.711
Duration of operation (min)	121 [99, 163]	123 [109, 161]	123 [99, 146]	0.812
Time to extubation (min)	12 [10, 15]	15 [10, 15]	10 [10, 15]	0.121
Insertion of laryngeal mask intubation	​	​	​	0.359
Very easy, n (%)	43 (86%)	47 (94%)	46 (92%)	​
Easy, n (%)	7 (14%)	3 (6%)	4 (8%)	​
First intubation attempt, n (%)	100 (100%)	100 (100%)	100 (100%)	1.000
Repositioning during surgery, n (%)	0 (0%)	0 (0%)	0 (0%)	1.000
Convert to tracheal intubation, n (%)	0 (0%)	0 (0%)	0 (0%)	1.000
Blood stain on laryngeal mask, n (%)	4 (8%)	3 (6%)	4 (8%)	0.907
Hypertension after gargle, n (%)	0 (0%)	1 (2%)	2 (4%)	0.360
Tachycardia after gargle, n (%)	0 (0%)	1 (2%)	3 (6%)	0.166
Intraoperative SpO_2_ < 90%, n (%)	0 (0%)	0 (0%)	0 (0%)	1.000
Peak airway pressure (cmH_2_O)				
3 min post-anesthesia induction	13.9 ± 2.09	14.1 ± 2.7	14.6 ± 2.5	0.214
30 min post-intubation	21.3 ± 3.2	21.1 ± 3.5	22.5 ± 4.2	0.128
60 min post-intubation	21.1 ± 3.7	21.5 ± 3.7	23.2 ± 4.2	0.053
90 min post-intubation	21.8 ± 3.3	21.6 ± 4.5	22.7 ± 3.6	0.567
120 min post-intubation	21.9 ± 3.7	22.9 ± 4.0	22.7 ± 3.0	0.480
Before extubation	16.6 ± 3.1	16.0 ± 3.1	17.5 ± 3.6	0.097
End-tidal carbon dioxide partial pressure (mmHg)				
3 min post-anesthesia induction	37.2 ± 3.8	37.6 ± 2.8	37.5 ± 3.2	0.823
30 min post-intubation	43.4 ± 4.3	43.5 ± 3.5	43.7 ± 3.9	0.908
60 min post-intubation	43.1 ± 4.7	44.4 ± 4.1	44.0 ± 3.9	0.296
90 min post-intubation	43.5 ± 3.9	44.3 ± 4.0	43.3 ± 3.7	0.670
120 min post-intubation	43.9 ± 4.6	44.0 ± 5.0	43.5 ± 5.1	0.868
Before extubation	42.0 ± 4.7	42.0 ± 4.5	41.0 ± 4.0	0.782

Data are presented as median [IQR], number (%), and mean ± SD. One-way analysis of variance (ANOVA) was used for continuous variables with normal distribution, the Kruskal–Wallis test for continuous variables with non-normal distribution, and chi-square tests for categorical variables.

## 4 Discussion

After exploring the efficacy, safety, and optimal dosage of esketamine gargle for preventing POST following laryngeal mask airway insertion, this study found that preoperative gargling with 25 mg and 50 mg of esketamine significantly reduced the incidence of POST without increasing the incidence of adverse events.

Tracheal intubation is currently acknowledged as the standard operational approach for airway safety. Owing to the advancement of supraglottic ventilation devices, laryngeal mask airways have gradually supplanted tracheal intubation for airway management in certain surgical procedures. Their advantages include no direct contact with the tracheal mucosa, no requirement for laryngoscopic exposure, and a reduced risk of complications such as hypoxemia and pulmonary infection (El-Boghdadly et al., 2016). Karaaslan E et al. (2021), in their comparison of the laryngeal mask airway and tracheal intubation for airway safety in nasal surgery, found that the laryngeal mask airway not only reduced the incidence of bleeding at the glottis, proximal trachea ([1–3] vs. [1–4]; *P* = 0.004), and distal trachea ([1–2] vs. [1–4]; *P* = 0.034), but also reduced the incidence and severity of POST at 2 h (*P* = 0.003), 6 h (*P* = 0.017), and 12 h (*P* < 0.001) postoperatively. Therefore, in the context of laparoscopic abdominal surgery, where the use of laryngeal mask airway is predominant and a large number of procedures are carried out, it is of significant clinical importance to explore interventions (such as esketamine gargling) and determine the optimal dosage to prevent POST.

Jaensson et al. (2024) conducted a comparison of the occurrence, location, and hoarseness of POST within 24 h after non - oral or non - pharyngeal area surgery among 112 male and 185 female patients. In cases where patients experienced POST, they were followed up at 48, 72, and 96 h after surgery until the symptoms subsided. The findings indicated that there was no significant gender - related difference in the incidence of POST and hoarseness following the use of a laryngeal mask airway and tracheal intubation. However, the incidence of POST (32% vs. 19%, *P* = 0.012) and hoarseness (57% vs. 33%, *P* < 0.001) after tracheal intubation was significantly higher than that after the use of a laryngeal mask airway. In our study, the incidence of POST within 24 h postoperatively was 12%, which was consistent with the results of the aforementioned report.

Although diverse measures are effective in reducing the incidence and severity of POST, drug preventive treatment, considered the most crucial intervention, encompasses local application of anesthetics, glucocorticoids, and other medications to the throat, inhalation via nebulization, intravenous and rectal administration of non-steroidal anti-inflammatory drugs, as well as the utilization of NMDA receptor antagonists (Wang et al., 2021; Assefa et al., 2022; Kuriyama et al., 2019). Magnesium sulfate, ketamine, and other NMDA receptor antagonists can also effectively reduce the incidence and severity of POST after tracheal intubation (Teymourian et al., 2015). Segaran et al. (2018) compared the administration of a magnesium-containing mouthwash (20 mg/kg) and a ketamine-containing mouthwash (0.5 mg/kg) 15 min preoperatively. They found that both drugs could reduce the incidence and severity of POST at 2 h, 4 h, and 24 h after appendectomy. The mechanism is attributed to the analgesic effect on the NMDA receptors in the pharyngeal mucosa and the inhibition of inflammatory effects resulting from pharyngeal mucosal injury and edema (Ittoop et al., 2023; Zhang et al., 2024; Puri et al., 2022).

A meta-analysis involving over 3,000 patients demonstrated that gargling (20–50 mg), nebulization inhalation (0.5–1.5 mg/kg), or local application (50 mg) of ketamine were all able to reduce the incidence of POST 24 h after tracheal intubation (RR 0.45; 95% CI: 0.37–0.54; *P* < 0.001) (Kuriyama et al., 2020). Park et al. (2010) administered an intravenous bolus of 0.5 mg/kg ketamine, followed by a low - dose maintenance infusion at a rate of 10 μg/kg/min. They found that this treatment protocol did not reduce the incidence or severity of POST within 24 h postoperatively. This finding implies that the mechanism of action of NMDA receptor antagonists is restricted to local effects. Therefore, the exploration of the administration method and dosage is of great importance for alleviating POST.

Liang et al. (2023) employed 50 mg of esketamine gargle, in comparison to a saline solution, to prevent POST in patients undergoing elective thoracic surgery following double-lumen tube intubation and found that esketamine significantly reduced the incidence of POST (12.9% vs. 47.1%, *P* < 0.001). Zhang et al. (2026) administered a nebulized inhalation of 5 mL of a 50-mg esketamine solution prior to thoracolumbar spine surgery. As a result, the incidence and severity of POST were significantly reduced at 2, 4, 6, 12, and 24 h postoperatively (all *P* < 0.05). Our results also lend support to the aforementioned findings, demonstrating that esketamine is both effective and safe in preventing POST. Given that our results indicate that the 25 mg and 50 mg doses seem to yield similar effects, we recommend the 25 mg dose.

In our study, insertion of the laryngeal mask airway was performed easily, and no conversions to endotracheal intubation occurred during the operation. The intraoperative peak airway pressure and PetCO_2_ levels were maintained within acceptable ranges at various time points. Despite the relatively longer operation and anesthesia durations for all three groups of patients, this still suggests that the laryngeal mask airway has a favorable application effect. However, even after gargling with esketamine in our study, there were still patients who experience hypertension and tachycardia, mainly in the 50 mg group. Subsequently, the aforementioned cardiovascular adverse events returned to normal within a relatively short period. Liang et al. (2023) administered 50 mg of esketamine gargle and found that no patients experienced adverse cardiovascular events. It cannot be excluded that factors such as the patient’s nervousness may have had an impact, and the sample size of our study is relatively small, additional large - sample trials may be required to comprehensively evaluate the impact of esketamine gargling on the cardiovascular system and further assess its safety in preventing POST.

This study has some limitations. First, the dose of esketamine gargle was not adjusted in accordance with the patients’ weight, which may have an impact on its preventive effect against POST. Second, a larger dose of esketamine gargle was not explored, taking into account the hemodynamic instability, such as hypertension and tachycardia, induced by sympathetic activation, which could pose risks to the patients’ safety. Third, female patients exhibit a higher incidence of POST following general anesthesia; therefore, restricting surgical studies to female patients alone may impede the comprehensive evaluation of interventions for POST during laryngeal mask airway ventilation. Finally, the mental complications caused by esketamine were not adequately evaluated, which might restrict its further promotion and application.

In conclusion, esketamine gargle is both effective and safe for preventing POST in patients undergoing laparoscopic abdominal surgery subsequent to laryngeal mask airway insertion. Among the two doses of 25 mg and 50 mg, 25 mg is the recommended dose.

## Data Availability

The original contributions presented in the study are included in the article/supplementary material, further inquiries can be directed to the corresponding author.
